# Antifungal Activity of Amphotericin B Conjugated to Nanosized Magnetite in the Treatment of Paracoccidioidomycosis

**DOI:** 10.1371/journal.pntd.0004754

**Published:** 2016-06-15

**Authors:** Camila Arruda Saldanha, Mônica Pereira Garcia, Diego Cesar Iocca, Luciana Guilherme Rebelo, Ana Camila Oliveira Souza, Anamélia Lorenzetti Bocca, Maria de Fátima Menezes Almeida Santos, Paulo Cesar Morais, Ricardo Bentes Azevedo

**Affiliations:** 1 Universidade de Brasília, Instituto de Ciências Biológicas, Departamento de Genética e Morfologia, Brasília, Distrito Federal, Brazil; 2 Universidade Estadual de Goiás, Unidade de Ciências Exatas e Tecnologia, Anápolis, Goiás, Brazil; 3 Universidade de Brasília, Instituto de Ciências Biológicas, Departamento de Biologia Celular, Brasília, Distrito Federal, Brazil; 4 Universidade de Brasília, Instituto de Física, Núcleo de Física Aplicada, Brasília, Distrito Federal, Brazil; University of California San Diego School of Medicine, UNITED STATES

## Abstract

This study reports on *in vitro* and *in vivo* tests that sought to assess the antifungal activity of a newly developed magnetic carrier system comprising amphotericin B loaded onto the surface of pre-coated (with a double-layer of lauric acid) magnetite nanoparticles. The *in vitro* tests compared two drugs; i.e., this newly developed form and free amphotericin B. We found that this nanocomplex exhibited antifungal activity without cytotoxicity to human urinary cells and with low cytotoxicity to peritoneal macrophages. We also evaluated the efficacy of the nanocomplex in experimental paracoccidioidomycosis. BALB/c mice were intratracheally infected with *Paracoccidioides brasiliensis* and treated with the compound for 30 or 60 days beginning the day after infection. The newly developed amphotericin B coupled with magnetic nanoparticles was effective against experimental paracoccidioidomycosis, and it did not induce clinical, biochemical or histopathological alterations. The nanocomplex also did not induce genotoxic effects in bone marrow cells. Therefore, it is reasonable to believe that amphotericin B coupled to magnetic nanoparticles and stabilized with bilayer lauric acid is a promising nanotool for the treatment of the experimental paracoccidioidomycosis because it exhibited antifungal activity that was similar to that of free amphotericin B, did not induce adverse effects in therapeutic doses and allowed for a reduction in the number of applications.

## Author Summary

Lung fungal infections are caused by pathogens inhaled as spores which convert into invasive yeast forms in the lungs. This type of infection can spread to other sites in the body through the blood and lymphatic systems, sometimes leading to ulcerations and skin lesions. The drug of choice for treatment is Amphotericin B (AmB). AmB is a typical polyene with broad-spectrum antifungal activity that encounters some use limitations because of its side effects. We developed a magnetic carrier nanocomplex comprising of amphotericin B loaded onto the surface of magnetite nanoparticles pre-coated with a double-layer of lauric acid. We evaluated this approach for its antifungal activity against *Paracoccidioides brasiliensis* (strain Pb18) and its cytotoxicity in mammalian cell culture. We found that this nanocomplex exhibited antifungal activity without cytotoxicity to human urinary cells and low cytotoxicity to peritoneal macrophages. *In vivo*, the nanocomplex did not induce genotoxic effects in bone marrow cells and was effective against experimental paracoccidioidomycosis without inducing clinical, biochemical or histopathological alterations.

## Introduction

Paracoccidioidomycosis (PCM) is a systemic fungal infection caused by *Paracoccidioides brasiliensis* and *Paracoccidioides lutzii* [1]. PCM represents the most important systemic mycosis in Central and South America. PCM significantly affects public health primarily targeting rural workers with limited access to the health care. Like many fungal infections, the *P*. *brasiliensis* pathogen is inhaled as spores and then germinates into the invasive yeast form in the lungs. The infection spreads to other sites through the blood and lymphatic systems, such as the oral mucous membranes and skin of the face, to cause several types of lesions [1–4]. Although PMC is seldom observed as an opportunistic infection, PCM occasionally occurs in immune-compromised patients.

Amphotericin B (AmB) is a typical polyene with broad-spectrum antifungal activity and is the drug of choice for the treatment of severe PCM or following the development of resistance to other first line drugs. Unfortunately, AmB causes acute side effects (primarily kidney problems) following intravenous administration, which limits its clinical use [5–8]. Therefore, to improve the therapeutic index of AmB and to reduce its toxicity for use in humans, new technological approaches have been employed in the engineering of recent formulations and drug delivery; these approaches include lipid-based preparations such as AmB lipid complex, AmB colloidal suspension, liposomal AmB, and AmB polymer [9–11]. Unfortunately, the clinical use of the new formulations is limited due to cost, preventing broad access for patients who depend upon public health care in many countries. As such, the development of new, affordable and effective antifungal delivery formulation with minimum cytotoxicity to human cells would be beneficial to patient health and economically beneficial.

Nanobiotechnology has already begun to have important effects of healthcare. Superparamagnetic iron oxide (SPIO) particles, which are a typical class of nanosized magnetic material, have been considered to be attractive magnetic probes for biological imaging and therapeutic applications [12, 13]. The use of SPIO in drug delivery vehicles must address issues such as drug-loading capacity, desired release profile, aqueous dispersion stability, biocompatibility with cells and tissue, and retention of magnetic properties after interaction with macromolecules or modification via chemical reactions. Magnetic fluid (MF) samples primarily consist of SPIO particles stably suspended in a carrier fluid and are of great importance due to their scientific and biotechnological potentials. SPIO particles, particularly magnetite (Fe_3_O_4_) and maghemite (γ-Fe_2_O_3_), are by far the most widely used magnetic nanoparticles (MNPs) in biological and medical applications [14]. The magnetic properties of MF samples and the huge variety of MNP surface functionalizations, which allow for specificity and biocompatibility while preventing agglomeration and precipitation of the MNPs in suspension, provide opportunities for applications in different areas [15]. For example, nanosized particles engineered to act as drug delivery systems that target the lungs have been extensively studied [16–22].

To investigate whether this approach reduces the side effects of AmB while simultaneously providing controlled drug-delivery system, a nanosized magnetite surface-functionalized MF sample with a bilayer of lauric acid conjugated with AmB (MFLB-AmB) was developed. The present study aimed to evaluate this novel MFLB-AmB complex in terms of its antifungal activity against *Paracoccidioides brasiliensis* (strain Pb18) and its cytotoxicity using mammalian cells. Additionally, this study aimed to evaluate the acute efficacy of MFLB-AmB in the treatment of an intratracheal infection of paracoccidioidomycosis induced in mice.

## Methods

### Sample preparation

Magnetite (Fe_3_O_4_) nanoparticles were synthesized by a chemical co-precipitation reaction in alkaline medium using an aqueous solution of Fe(II) and Fe(III) ions following a procedure that has been described in the literature [23]. Briefly, the two aqueous-based solutions were combined in a beaker, and concentrated ammonium hydroxide solution was added while stirring. The black precipitate (Fe_3_O_4_) was separated from solution using a permanent magnet. Then, lauric acid (LA; Sigma-Aldrich, St. Louis, MO, USA) was added to the black precipitate for surface functionalization of the freshly precipitated magnetite nanoparticles. The mixture was then heated at 90°C while stirring and water was added to adjust the volume. The magnetite nanoparticles were then spontaneously dispersed into the aqueous medium to yield a water-based MF sample. Finally, the purified MF sample was autoclaved for sterilization at 121°C for 20 min. The stock MF sample, containing the LA bilayer-coated nanoparticles suspended in aqueous medium, was labeled MFLB. The nanoparticle concentration within the MFLB sample was estimated to be approximately 2.3×10^15^ particle/mL, whereas the iron concentration was 1.52 mg/mL. The association of AmB (Sigma-Aldrich, St. Louis, MO, USA) onto the LA bilayer-coated nanoparticles was performed using a protocol similar to one that has been described in the literature [24] in which AmB was dispersed in dimethyl sulfoxide (DMSO; Mallinckrodt, USA) and mixed with the MFLB stock sample while stirring. The resulting complex material system (MFLB-AmB) containing the AmB adsorbed onto the LA-bilayer coated magnetite was separated from the free drug excess medium by magnetic separation and re-dispersed in sterile water. The magnetic nanoparticle concentration within the MFLB-AmB complex was estimated to be approximately 2.6×10^15^ particle/mL, and the iron concentration was found to be 1.74 mg/mL. The adsorbed AmB concentration was indirectly determined by quantifying the excess drug (free AmB) using UV-Vis measurements (Spectra Max M2, Molecular Device, USA) tuned to 411 nm. The measured AmB content incorporated in the MFLB-AmB complex was found to be 1.02 mg/mL. Notably, the MFLB-AmB complex incorporated iron (1.74 mg/mL) and AmB (1.02 mg/mL) within the same range of values per unit volume. Similarly, the iron content in the MFLB sample (1.52 mg/mL) fell in the same range. Indeed, the physical-chemical characteristics of the MFLB-AmB complex have been investigated by Santos et al. [25] who reported on the molecular anchoring of AmB onto the pre-coated magnetite nanoparticles. Briefly, the average particle diameters (standard deviation) associated with samples of MFLB and MFLB-AmB as assessed via curve-fitting of the particle size histograms obtained from transmission electron microscopy (TEM) were 6.0 (0.36) and 7.1 (0.28) nm, respectively. The difference we observed in the values of the deposited (TEM sample holder) average particle diameters (6.0 and 7.1 nm) is consistent with the values obtained for the hydrodynamic diameters of particles suspended within samples MFLB (77.7 nm) and MFLB-AmB (84.8 nm) as estimated from dynamic light scattering measurements. The larger hydrodynamic diameter observed for the MFLB-AmB sample was primarily due to the adsorption of AmB onto the nanoparticle’s LA pre-coating layer. Notably, although the magnetic nanoparticle, iron, and AmB concentrations within the MFLB-AmB complex were similar to those of the sample used in the *in vitro* assays, the hydrodynamic diameter observed for the MFLB-AmB sample used in the *in vivo* assays was greater (137.0 nm).

### Cell and culture description

*Paracoccidioides brasiliensis* strain 18 (Pb18) was obtained from the fungal collection of the Laboratory of Molecular Biology, Institute of Biological Science, University of Brasília (Brazil). The Pb18 strain was cultured in liquid YPD medium (w/v: 2% peptone, 1% yeast extract, 2% glucose) at 36°C in a rotary shaker (220 rpm). After 5 days of growth, a suspension of *P*. *brasiliensis* cells was prepared at a concentration of 1×10^7^ viable cells/mL. Viability was determined with Janus Green B vital dye (Merck, Darmstadt, Germany) and was always greater than 80%. Pb18 was then used to test the antifungal activity of the MFLB-AmB complex.

Human mesangial cells (a renal cell line) were obtained from the American Type Culture Collection (ATCC, USA) and maintained in the Laboratory of Nanobiotechnology, University of Brasília (Brazil). These cells were grown at 37°C in a 5% CO_2_ atmosphere within Dulbecco's modified Eagle's medium (DMEM) (Gibco, New York, NY, USA) at a pH of 7.4 supplemented with 10% fetal calf serum (Life Technologies, Brasil), 100 U/mL penicillin and 100 μg/mL streptomycin.

Peritoneal cells were obtained from the peritoneal cavities of 6-week-old BALB/c mice. Briefly, the mice were anesthetized in a chamber with isoflurane and killed by CO_2_ gas. A small incision was made in the abdominal skin while keeping the peritoneal membrane intact, and 20 mL of sterile, cold phosphate buffered saline (PBS, pH 7.4) was gently injected using a 25-mL syringe with a 20-gauge needle. Soft abdominal massage was performed to wash (lavage) the intraperitoneal cavity. Using a Pasteur pipette, the lavage was slowly aspirated and centrifuged at 1000 rpm for 10 min. The pellet was then washed three times with red blood cell lysis buffer. The harvested peritoneal cells of the final pellet were plated with RPMI 1640 medium (Invitrogen) with 10% fetal bovine serum (FBS; Laborclin, Pinhais, PR, Brazil) and 1% penicillin/streptomycin and incubated at 37°C under 5% CO_2_ for 3 hours. Non-adherent cells were removed by washing with PBS, and adherent cells, i.e., macrophages, were re-incubated.

### Evaluation of the in vitro antifungal activity

The antifungal activity of the MFLB-AmB complex was evaluated by assessing the minimum inhibitory concentration (MIC) index. The MIC was defined as the lowest drug concentration for which a 90% inhibition of growth in colony-forming units (CFUs). Fungal cell suspensions (2×10^4^ cells/100 μL) were incubated in RPMI 1640 medium supplemented with the MFLB-AmB complex with AmB concentrations ranging from 0.125 to 8.0 μg/mL at 37°C. After 72 hours, the cell suspensions were removed from the media and transferred to Petri plates containing brain heart infusion medium (BHI; HiMedia Laboratories, Mumbai, India) supplemented with 4% (v/v) horse serum (Gibco, New York, NY, USA), 5% (v/v) of the supernatant from the culture filtrate of the isolate Pb192 and 40 mg/L gentamycin (Schering-Plough, USA). The filtrate was prepared according to the methodology described by Singer-Vermes et al. [26]. The fungal cells were then incubated for five days at 37°C in a rotary shaker (220 rpm). The control groups consisted of untreated fungal cells and fungal cells treated with the MFLB sample only. The MFLB content is described in terms of iron concentration and ranged from 0.21 to 13.64 μg/mL.

### In vitro MTT cytotoxicity assay

To evaluate the cytotoxicity of the MFLB-AmB complex using mammalian cells, we performed an MTT assay as described by Wasan et al. [27]. The colorimetric MTT assay is based on the capability of viable cells to reduce the yellow product MTT (3-(4,5)-dimethylthiazol-2-yl-2,5-diphenyltetrazolium bromide) to a blue product (formazan) via a dye reduction reaction that occurs only in the mitochondria of viable cells. Blue formazan crystals can be dissolved in organic solvent, and the concentration can be quantified via spectrophotometric analysis. Thus, the number of living cells is directly proportional to the intensity of the blue color.

Human mesangial cells and murine peritoneal macrophages (5×10^4^ cells each) were treated in culture medium supplemented with AmB alone or with the MFLB-AmB complex in which the AmB concentrations ranged from 0.25 to 1.0 μg/mL. The treatment was performed at 37°C for 6, 12 or 24 hours. The control groups consisted of untreated cells and cells treated with MFLB only, with iron concentrations in the range of 0.42 to 204.70 μg/mL were quantified for the latter. After treatment, the cells were incubated with MTT (0.5 mg/mL- Invitrogen, Grant Island, NY, USA) for 4 hours at 37°C. Formazan crystals were dissolved in 200 μL of dimethyl sulfoxide (DMSO). Cell viability was assessed by measuring the absorbance at 590 nm using a microplate reader (SpectraMax M2, Molecular Device, USA). The results are expressed as LC_50_s, the concentration required to reduce the cell population by half in a given time. The LC_50_ values were estimated following probit analyses [28].

### Animals and in vivo experimental design

#### Ethics statement

All animal handling and procedures were performed according to the Guide for the Care and Use of Laboratory Animals published by the National Institutes of Health, USA, and were approved by the Comitê de Ética no Uso Animal–CEUA (Animal Ethics Committee of the University of Brasilia),–UnBDoc number 12155/2007.

Female BALB/c mice (6–8 weeks old) weighing 20–25 g were obtained from the Central Animal Facility of the University of Brasilia (Brasília, Brazil). The animals were acclimatized to the laboratory conditions for two weeks prior to the initiation of the study. They were housed in plastic cages (6 animals/cage) at room temperature (20 ± 2°C) on a 12-hour light/dark cycle with the lights on at 6 a.m. and provided free access to food and filtered water. After acclimatizing, the animals were anesthetized by intraperitoneal injection of ketamine (80 mg/kg) and xylazine (10 mg/kg) in the same syringe following a final volume of 0.1 mL/30 g and their necks were hyperextended to expose the trachea at the thyroid level. Animals were then infected intratracheally with 3 × 10^6^ *P*. *brasiliensis* yeast cells, according to Fernandes and coworkers [29]. The establishment of disease was confirmed in one animal 30 days after infection, with lung histopathology revealing compact granulomas containing fungal cells, polymorphonuclear leukocyte aggregates, and fibrous tissue.

After inoculation with the pathogens, animals were randomly divided into the following four experimental groups (n = 10) as detailed in Table 1: infected mice treated with PBS (positive control: PC group); infected mice treated with MFLB (MFLB group); infected mice treated with AmB (AmB group); and infected mice treated with MFLB-AmB (MFLB-AmB group). The treatment began 24 hours after inoculation with the pathogen. The MFLB, MFLB-AmB, and PBS were administered every 3 days via nasal instillation, whereas AmB was administered daily via intraperitoneal injection. The treatments lasted 30 or 60 days. Non-infected animals treated with PBS served as the negative control (NC) group.

**Table 1:** Experimental design.

Group (n = 10)			Treatment	
	PBS(μL)*	MFLB (μg/μL)*	AmB (μg/μL)**	MFLB-AmB (μg/μL)*
Animals not infected (NC group)	80	-	_	-
Animals with infection (PC group)	80	-	_	-
Animals with infection (AmB group)	_	-	40/100	-
Animals with infection (MFLB group)	_	80	-	-
Animals with infection (MFLB-AmB group)	_	-	-	60,8/80

Female BALB/c mice uninfected (NC–negative control) and infected with *Paracoccidioides brasiliensis*, treated with PBS (PC–positive control), AmB (Amphotericin B), MFLB-AmB (nanosized magnetite surface-functionalized with a bilayer of lauric acid conjugated with Amphotericin B) and MFLB (nanosized magnetite surface-functionalized with a bilayer of lauric acid).

* nasal instillation

** intraperitoneal

During the experimental time, mice were clinically examined and photographed for clinical alterations. Additionally, the animals were monitored for weight gain or loss.

### Procedures and measurements

Twenty-four hours after each treatment, the animals were anesthetized with a mixture of xylazine and ketamine according to the method described above, the body weights were measured, and blood samples (1 mL) were collected via the ocular capillary and animals were killed by cervical dislocation according to the AVMA Guidelines on Euthanasia [30]. The spleen, liver, kidneys and lungs were removed for morphology analyses. The lungs were also removed to determine the fungal burden and to quantify cytokine levels. Bone marrow cells were collected to measure the rate of DNA fragmentation.

### Biochemical and enzymatic analysis of blood

Blood samples were collected to assess the levels of total creatinine and urea as kidney function markers and transferrin, aspartate aminotransferase (AST) and alanine aminotransferase (ALT) as liver function markers. The analyses were run on an ADVIA 2400 (Siemens, USA) automated chemistry analyzer using the appropriate ADVIA chemistry reagents and protocols. Total creatinine, urea, and transferrin were measured via colorimetric assays, and AST and ALT were measured via optimized kinetic methods.

### Histopathology analysis

To perform the morphological analyses, fragments of the organs were fixed with buffered paraformaldehyde (4%) at room temperature for three hours, transferred to 70% ethanol, embedded in paraffin using an automatic tissue processor (OMA DM-40, São Paulo, Brazil), and cut to 5-μm thick slices on a Leica RM2235 manual microtome (Leica Microsystems, Nussloch, Germany). The slides were stained with hematoxylin-eosin (H&E) for histological analyses. All histological sections were photographed with a CCD camera with Axio Vision 40v 4.6.1.0 software in a Zeiss Axioskop light microscope (Zeiss, Germany).

### Lung tissue preparations to determine fungal burdens and for cytokine assays

To determine the fungal burdens and quantify cytokines, such as interferon-gamma (IFN- γ), interleukin-4 (IL-4), 10 (IL-10), and 12 (IL-12), in the lungs, lung fragments were weighed, macerated, and homogenized in 1 mL cold, sterile PBS.

Briefly, for the determinations of the fungal burdens, aliquots of the homogenized pulmonary tissue were plated on Petri dishes containing Brain Heart Infusion medium (BHI, Acumedia, USA) supplemented with horse serum 4% (v/v), 5% (v/v) supernatant isolated from the filtrated culture PB192, and 40 mg/L gentamicin. The colony-forming units (CFUs) were counted seven days after plating and incubation at 37°C.

For the cytokine assays, aliquots of the homogenized tissue were centrifuged at 400 g for 20 min, and the cytokine analyses were performed on the supernatants. The Bradford method was used for total protein quantification. The experimental protocols for the interferon-gamma (IFN- γ), interleukin-4 (IL-4), 10 (IL-10), and 12 (IL-12) analyses were determined by manufacturers of the ELISA kits (e-Bioscience and BD, USA). These cytokines were quantified because the presence of IFN- γ and IL-12 are associated with mild forms of disease in models of pulmonary infection, and the presence of IL-10 and IL-4 are associated with the severe forms of the disease [31].

### DNA fragmentation measures

Bone marrow cells were collected from the femur bone and washed with 1 mL of fetal calf serum. The collected material was centrifuged at 250 g for three minutes. The resulting pellet was resuspended in cold ethanol (70%) to fix the cells. After 24 hours, the material was centrifuged at 250 g for three minutes and washed in PBS; resuspended in a 200 μL lysis buffer (0.1% sodium citrate [Invitrogen, USA], 0.1% Triton X-100 [Merck, Germany]) containing propidium iodide (20 μg/mL) in PBS (Sigma, USA). After 30 minutes and constant protection from light, the DNA fragmentation was measured at 560–580 nm in a flow cytometer (BD FACSCalibur, USA).

### Statistical analyses

The results are expressed as the means ± the SEMs of the different groups. The statistical analyses were performed by one-way analyses of variance (ANOVA), and Tukey’s tests (GraphPad PRISM 5.0, USA) were used when statistically significant differences were found. Differences were considered significant at P < 0.05.

## Results

**Fig 1:**
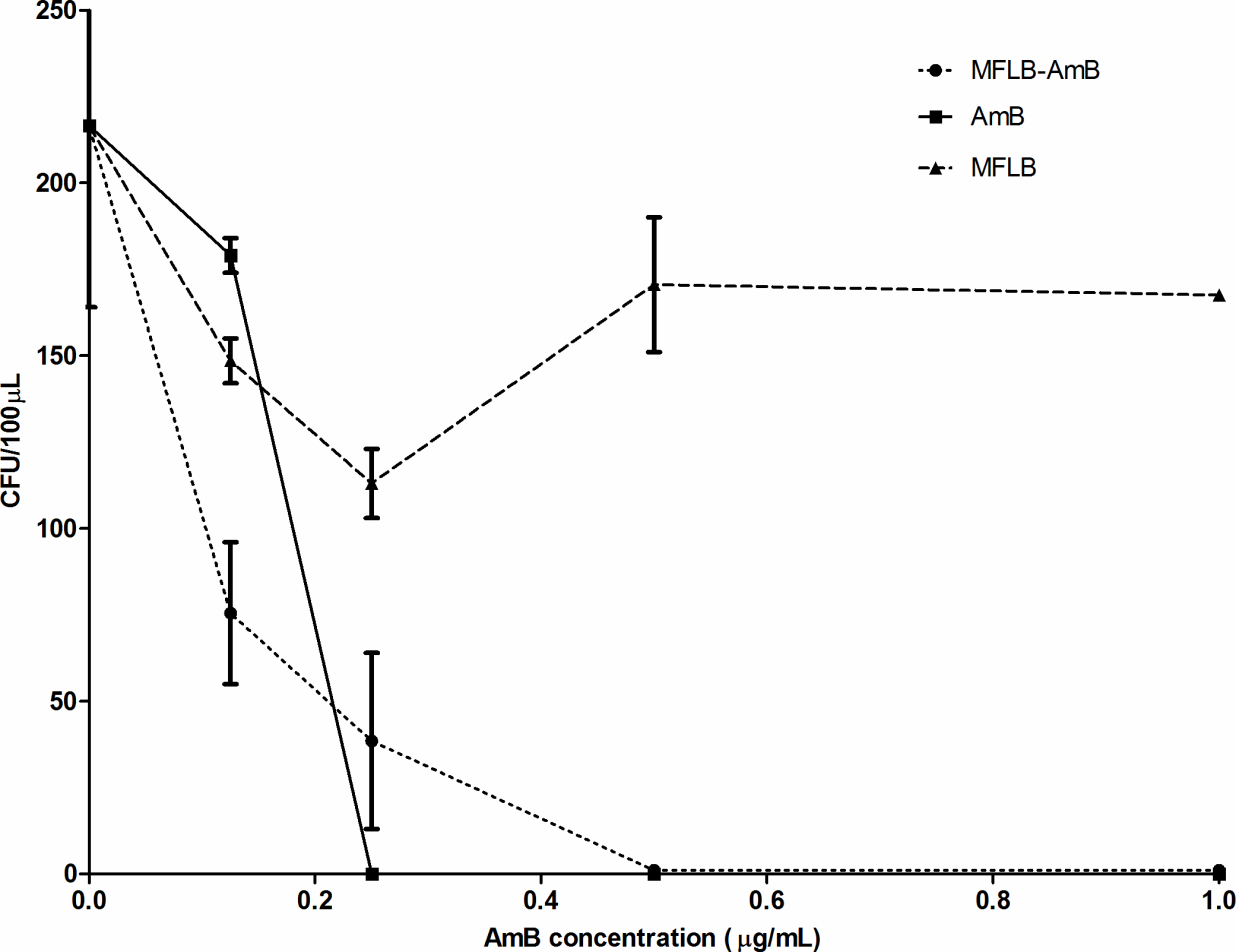
MIC determination of free AmB and MFLB-AmB against liquid suspensions of *Paracoccidioides brasiliensis*. Fungal burden as assessed by Colony Forming Units (CFU) in *Paracoccidioides brasiliensis* strain 18 (Pb18) (2 x 10^4^ cells/mL) while incubated in RPMI 1640 medium at 37°C for 72 hours with AmB or MFLB-AmB, as a function of amphotericin B, or MFLB, as a function of iron concentration. After 72 hour incubation, cell suspensions were plated on BHI (brain heart infusion) media, supplemented with 4% (v/v) of horse serum, 5% (v/v) of the supernatant from the culture filtrate of isolate Pb192 and 40 mg/L gentamycin, and then incubated for five days at 37°C in a rotary shaker (220 rpm).(AmB = amphotericin B, MFLB-AmB = nanosized magnetite surface-functionalized with a bilayer of lauric acid conjugated with amphotericin B, MFLB = nanosized magnetite surface-functionalized with a bilayer of lauric acid).

Antifungal activity against *Paracoccidoides brasiliensis* (Pb18 strain) was determined via colony-forming units (CFU) versus AmB concentration (μg/mL) added to the culture medium (Fig 1). The data displayed is corrected for untreated controls (the Pb18 strain incubated with RPMI 1640 medium only). The MIC indices for MFLB-AmB and AmB were 0.50 μg/mL and 0.25 μg/mL, respectively (Fig 1). In spite of the fact that the MIC for MFLB-AmB is the double of that observed with AmB, it is clear that the Amphotericin B when conjugated to magnetic nanoparticles stabilized with bilayer lauric acid maintains fungicidal activity.

**Fig 2:**
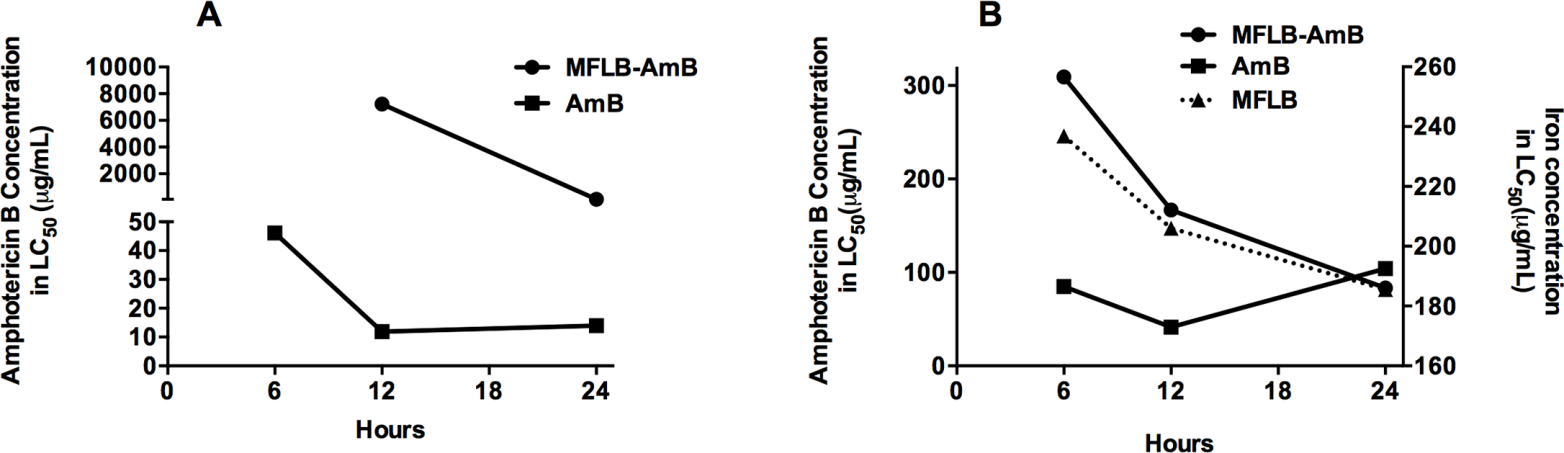
Cytotoxicity of AmB, MFLB-AmB or MFLB toward human mesangial cells and isolated mouse peritoneal macrophages. Concentration of amphotericin B (left vertical axis) in AmB or MFLB-AmB or iron concentration (right vertical axis–panel B) in MFLB required to reduce by half the cell population (LC_50_) at different time windows, A) human mesangial cells and B) mice peritoneal macrophage cells 5×10^4^ cells were treated in culture medium with AmB alone or with the MFLB-AmB complex rangin AmB concentration from 0.25 to 1.0 μg/mL at 37°C for 6, 12 or 24 hours. After indicated times, cells were incubated with MTT (0.5 mg/mL- Invitrogen, Grant Island, NY, USA) for 4 hours at 37°C. Formazan crystals were dissolved in 200 μL of dimethyl sulfoxide (DMSO). Cell viability was assessed by measuring the absorbance at 590 nm, using a microplate reader. LC_50_ values were estimated following Probit Analyses. (AmB = amphotericin B, MFLB-AmB = nanosized magnetite surface-functionalized with a bilayer of lauric acid conjugated with amphotericin B, MFLB = nanosized magnetite surface-functionalized with a bilayer of lauric acid treated at the same corresponding iron concentration of MFLB-AmB, ranging from 0.42 to 204.70 μg of Fe/mL).

The results of the LC_50_ tests of the MFLB-AmB nanocomplex are shown in Fig 2 using human mesangial cells (Fig 2A) and mice peritoneal macrophages (Fig 2B). AmB was included in the LC_50_ test for comparison. An LC_50_ test of MFLB with human mesangial cells was also included. AmB concentration is denoted on the left vertical axis (Fig 2A and 2B), whereas the iron concentration (for MFLB) is plotted on the right vertical axis (only in Fig 2B). After 6 hours of incubation with the MFLB-AmB nanocomplex, the human mesangial cells did not exhibit remarkable cell death, and no LC_50_ could be calculated. After 12 hours of incubation, the LC_50_ value for the MFLB-AmB complex (LC_50_ = 7225 μg/mL) was approximately six hundred times higher than the LC_50_ value for the free AmB (LC_50_ = 11.9 μg/mL). Although the LC_50_ value of the MFLB-AmB complex decreased to 89.98 μg/mL after 24 hours of incubation, it was still six-fold higher than the LC_50_ value of the free AmB (LC_50_ = 13.19 μg/mL) (Fig 2A). Regarding the peritoneal macrophages, after 6 and 12 hours incubation with the MFLB-AmB complex, we observed LC_50_ values of 309.3 and 166.7 μg/mL, respectively. Note that these values were four-fold higher than the LC_50_ values observed for free AmB, which were 85 μg/mL (after 6 hours incubation) and 41.6 μg/mL (after 12 hours incubation). However, after 24 hours of incubation with the peritoneal macrophages, the LC_50_ values of the MFLB-AmB complex (LC_50_ = 83.43 μg/mL) and free AmB (LC_50_ = 104 μg/mL) were roughly the same (Fig 2B).

**Fig 3:**
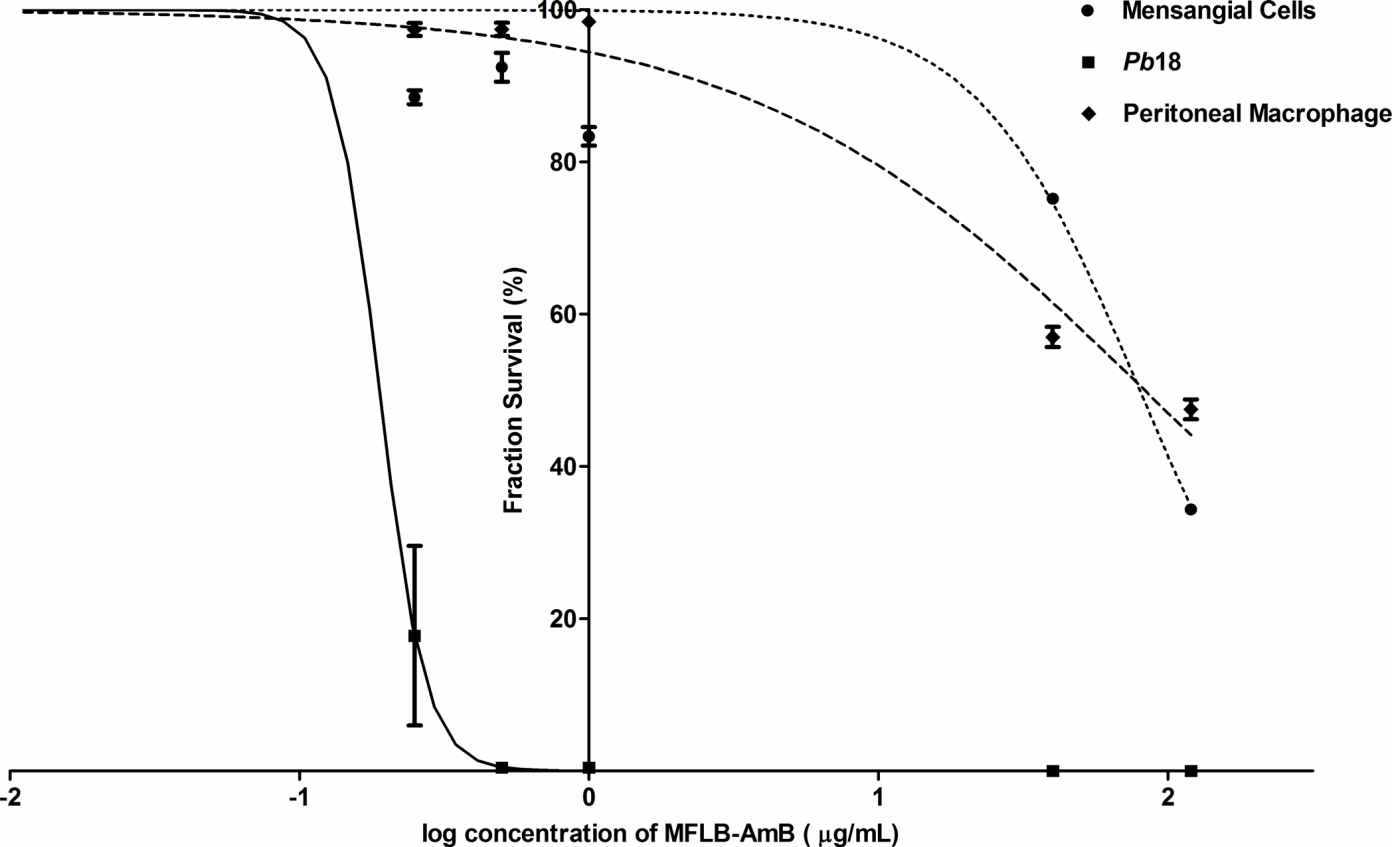
MFLB-AmB complex was more cytotoxic to fungal cells than to mammalian cells. Comparison of *in vitro* cytotoxicity of the MFLB-AmB (nanosized magnetite surface-functionalized with a bilayer of lauric acid conjugated with amphotericin B) against fungal (*Paracoccidoides brasiliensis* strain 18 –Pb18) and mammalian cells (human mesangial cells and peritoneal macrophage). Fraction survival of indicated cells versus log concentration of MFLB-AmB (μg/mL).

The MFLB-AmB complex was more cytotoxic to fungal cells than to mammalian cells (Fig 3). For the same MFLB-AmB complex concentration, the mesangial cell viability was higher than the macrophage viability. Additionally, the mesangial cells were more resistant at lower MFLB-AmB complex concentrations, although cytotoxicity was enhanced at higher MFLB-AmB concentrations (Fig 3).

**Fig 4:**
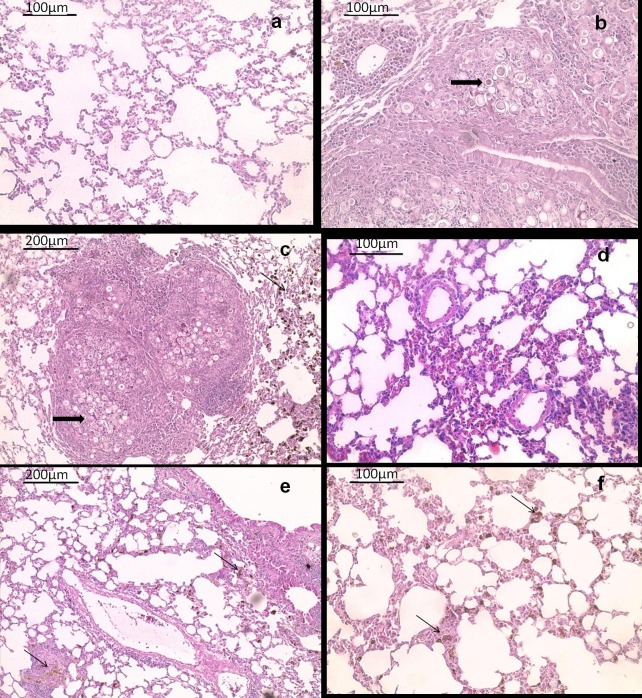
Histological appearance of female BALB/c mice lungs tissue hematoxylin and eosin-stained sections. Representative images are shown from (a) uninfected animal (b) infected animal with PBS treatment (c) infected animal treated with MFLB (d) infected animal treated with AmB and (e & f) infected animal treated with MFLB-AmB. Thick arrows in (b) and (c) indicate the presence of fungal cells inside granuloma. Asterisks in (b) indicate inflammatory focus; thin arrow in (c), (e) and (f) indicate nanoparticle aggregation. Portions of lungs were fixed in buffered paraformaldehyde 4% at room temperature for three hours followed by routine paraffin embedded procedure using an automatic tissue processor. Tissue sections of 5 μm of thickness were obtained with a manual microtome. (AmB = amphotericin B, MFLB-AmB = nanosized magnetite surface-functionalized with a bilayer of lauric acid conjugated with amphotericin B, MFLB = nanosized magnetite surface-functionalized with a bilayer of lauric acid; animals were infected with *Paracoccidioides brasiliensis*).

**Fig 5:**
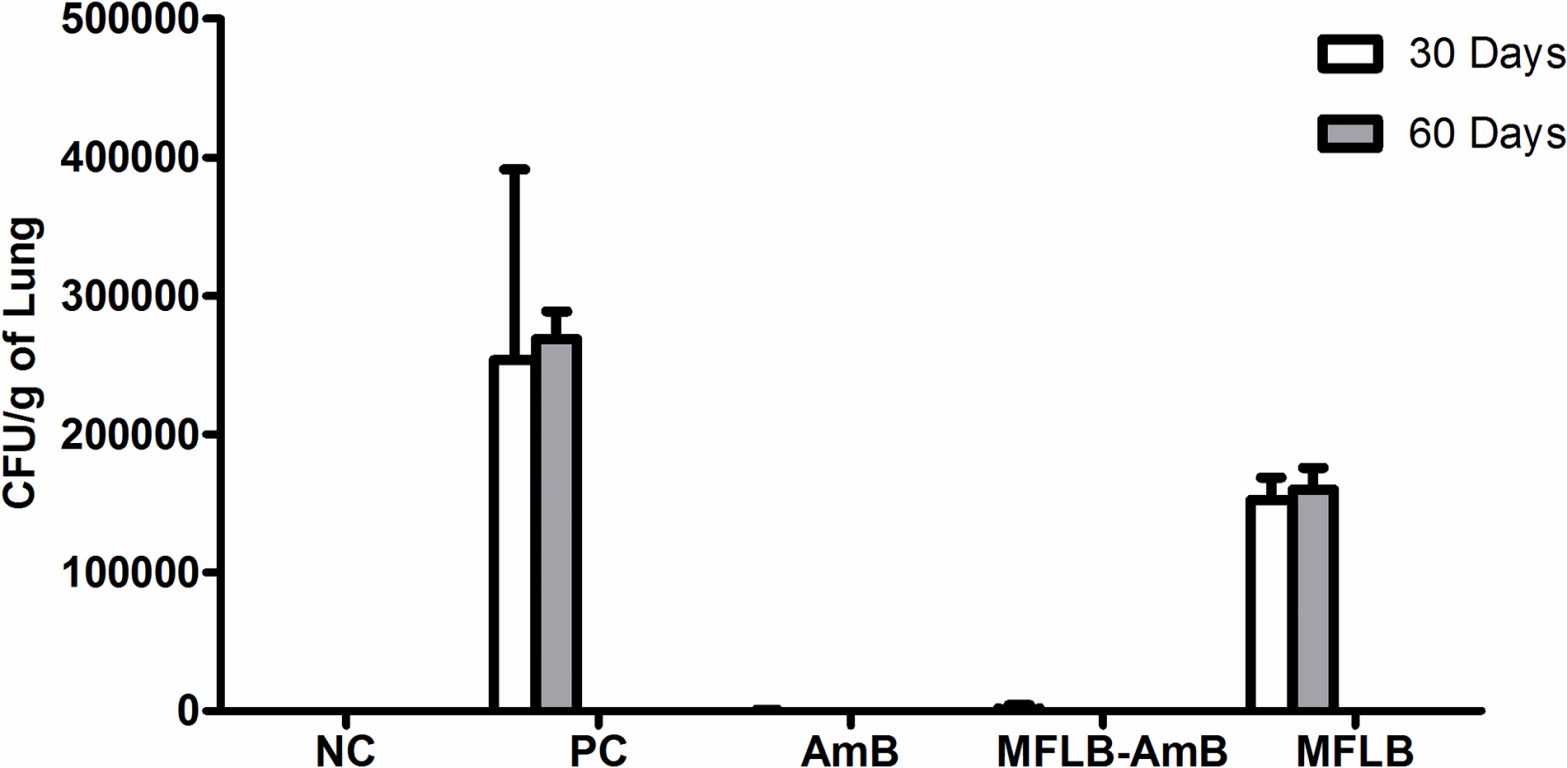
Fungal burden as assessed by CFU from mice lungs treated with AmB, MFLB-AmB, and MFLB. Lung fragments were weighed, macerated and homogenized in 1 mL cold, sterile PBS. Part of homogenized pulmonary tissue was plated on Petri dishes containing BHI (Brain Heart Infusion) medium, supplemented with 4% (v/v) horse serum, 5% (v/v) supernatant isolated from the infiltrated culture Pb192, and 40 mg/L gentamicin. CFU per gram of lung were determined seven days after plating with incubation at 37°C. Negative Control Group = animals uninfected; Positive Control, MFLB, AmB and MFLB-AmB Groups = animals infected with *Paracoccidioides brasiliensis*; (AmB = Amphotericin B; MFLB-AmB = nanosized magnetite surface-functionalized with a bilayer of lauric acid conjugated with AmB; MFLB = nanosized magnetite surface-functionalized with a bilayer of lauric acid).

The efficacy of MFLB-AmB against strain Pb18 was also investigated *in vivo* using a mouse model of paracoccidioidomycosis (PCM). H&E stained sections of lungs harvested from animals from the PC group (Fig 4B) and MFLB group (Fig 4C) had lungs with fungal cells with granulomas and inflammatory cells near the blood vessels and the bronchial tree. The lung tissue sections from the uninfected animals (NC group) exhibited typical appearances with no histological lesions (Fig 4A). Following acute treatment with the MFLB-AmB nanocomplex or free AmB,resulted in a 100% absence of detection in fungal load as observed via lung sections after 30 days of treatment (Fig 4D–4F) and CFU in lung homogenates after 60 days of treatment (Fig 5). However, the animals from the AmB groups exhibited inflammatory cells near the blood vessels and the bronchial tree (Fig 4D).

To evaluate the basis of the histological lesion category, we quantified cytokines such as interferon-gamma (IFN- γ), interleukin-4 (IL-4), 10 (IL-10), and 12 (IL-12) in the lung homogenates. Thirty days after treatment all interleukins levels reveal changes due to infection; while IFN-γ, IL-4, and IL-12 decreased (PC < NC), IL-10 increased (PC > NC). The two antifungal treatments (MFLB-AmB and AmB) showed different trends when compared with the PC (Table 2). The AmB treatment increased the levels of IFN-γ, IL-4 and IL-12 compared to PC mice (AmB > PC). MFLB-AmB treatment decreased the levels of IL-4 and IL-12 in an opposite direction compared to PC treatment (MFLB-AmB < PC) while keeping the IFN-γ level unchanged (MFLB-AmB = PC). As far as the IFN-γ level is concerned, MFLB did not change the effects of AmB while conjugated in one single structure (AmB = MFLB-AmB). However, compared to MFLB, the levels of IL-4 and IL-12 were reduced after treatment with AmB-MFLB (MFLB > MFLB-Amb), indicating that inclusion of MFLB into the MFLB-AmB complex does modulate the AmB alone treatment effects on IL-4 and IL-12 levels (Table 2).

**Table 2:** Cytokine levels, as determined by ELISA, in lung homogenates prepared from female BALB/c mice with AmB, MFLB-AmB, and MFLB.

group	IFN-γ (pg/mg)		IL-4 (pg/mg)		IL-10 (pg/mg)		IL-12 (pg/mg)	
	30 days	60 days	30 days	60 days	30 days	60 days	30 days	60 days
NC	919±179^B^	922±200^B^	200±14^B^	169±22^B^	12.3 ±2.4^B^	14.0 ±3.0^B^	1546±234^A^	1521±378^B^
PC	213±71^C^	2316±580^A^	90±12^C^	439±31^A^	64.5 ± 12.8^A^	167 ±5.4^A^	554±109^B^	2987±580^A^
AmB	1515±224^A^	426±172^C^	768±184^A^	200±47^B^	103.2±15.8^A^	205±113^A^	2132±323^A^	634±74^C^
MFLB-AmB	99±30^C^	919±288^B^	2.3±0.3^D^	265±78^B^	94.7±36.5^A^	70 ±4.2^A^	234±45^C^	1432±287^B^
MFLB	470±130^C^	922±341^B^	130±51^C^	254±40^B^	56.7±24.0^A^	123 ±27^A^	679±67^B^	1580±322

NC Group = animals uninfected; Positive Control, MFLB, AmB and MFLB-AmB Groups = animals infected with *Paracoccidoides brasiliensis*; AmB = amphotericin B; MFLB-AmB = nanosized magnetite surface-functionalized with a bilayer of lauric acid conjugated with AmB; MFLB = nanosized magnetite surface-functionalized with a bilayer of lauric acid

Different letters denote significant differences between treatments (Tukey, p < 0.05) inside the same column.

Sixty days after treatment the infected animals (PC) showed increased levels of all interleukins compared with uninfected animals (NC). Both antifungal agents (MFLB-AmB and AmB) reduced the IFN-γ, IL-4, and IL-12 levels compared with the PC (Table 2). However, we found that reduction of IFN-γ and IL-12 levels induced by the AmB treatment was significantly higher than that induced by the MFLB-AmB treatment. Comparison between treatments performed with MFLB-AmB and MFLB showed that levels of all interleukins did not differ (MFLB = MFLB-AmB), indicating that in the long term the presence of MFLB did not interfere with the AmB action (Table 2). The levels of IL-10 were not affected by any treatment (MFLB-AmB = AmB = PC), either after 30 or 60 days (Table 2).

We also evaluated whether the MFLB-AmB complex induced toxicity in the kidneys, liver, and spleen. The spleen, liver, and kidneys of the animals treated with the MFLB-AmB complex exhibited no morphologic abnormalities. Examination of urea and creatinine values as markers of kidney function and transferrin, aspartate aminotransferase (AST) and alanine aminotransferase (AST) as markers of liver functions were also performed. MFLB-AmB complex induced significant increases in urea levels after 60 days of treatment when compared to the NC group. The same occur in AmB and MFLB-AmB in urea levels after 60 days of treatment when compared to the PC group. (Table 3). MFLB induce a significant decrease in urea levels after 60 days of treatment when compared to NC group.

**Table 3:** Biochemical and enzymatic analysis of blood from female BALB/c mice after treatment with AmB, MFLB-AmB, and MFLB for 30 or 60 days.

Groups	Urea(mg/DL)		Creatinine (mg/DL)		AST (UI/L)		ALT (UI/L)		Transferrin	
	30 days	60 days	30 days	60 days	30 days	60 days	30 days	60 days	30 days	60 days
NC	49 ±7.4	57±6.6^C,B^	0.22±0.1	0.22±0.05	101±20.28^A^	83±3.31^A^	7±2.2	12.25±1.70	222±2.38^B^	220±3.7
PC	48±3.5	48±5.7^C^	0.3±0.1	0.23±0.05	128±24.84^A,B^	71±0.57^B^	10±1.6	6.66±2.51	227±3.60^A,B^	217±10.5
AmB	62±9	61±4.0^B^	0.24±0.1	0.23±0.11	133±22.65^A,B^	85±8.00^A,B^	10±0.8	10.33±2.51	225±12.9^A,B^	233±5.5
MFLB-AmB	53±8.7	79±1.0^A^	0.27±0.05	0.30±0	97±16.34^A,B^	114±2.30^A,B^	9±3.2	10.00±0	234±4.12^A^	232±0.6
MFLB	60±5.6	43±1.5^D^	0.30±0	0.30±0	69±0.57^B^	91±20.51^A,B^	12±1.7	9.66±0.57	226±5.85^A,B^	227±10.2

NC Group = animals uninfected; Positive Control, MFLB, AmB and MFLB-AmB Groups = animals infected with Paracoccidoides brasiliensis; AmB = amphotericin B; MFLB-AmB = nanosized magnetite surface-functionalized with a bilayer of lauric acid conjugated with amphotericin B; MFLB = nanosized magnetite surface-functionalized with a bilayer of lauric acid

Different letters denote significant differences between treatments (Tukey, p < 0.05) inside the same column. Treatments without lettering are not significantly different inside the same column.

The treatment AmB, MFLB-AmB or MFLB doesn’t show significantly different in creatinine and transferrin levels after 30 and 60 days when compared to NC and PC groups.

MFLB induce a significant decrease in AST levels after 30 days of treatment when compared to the NC group. But after 60 days of treatment that difference is not significant. The same occur in ALT levels with MFLB treatment, after 30 days of treatment induce significant increase the levels when compared to the NC group. But after 60 days of treatment that difference is not significant.

**Fig 6:**
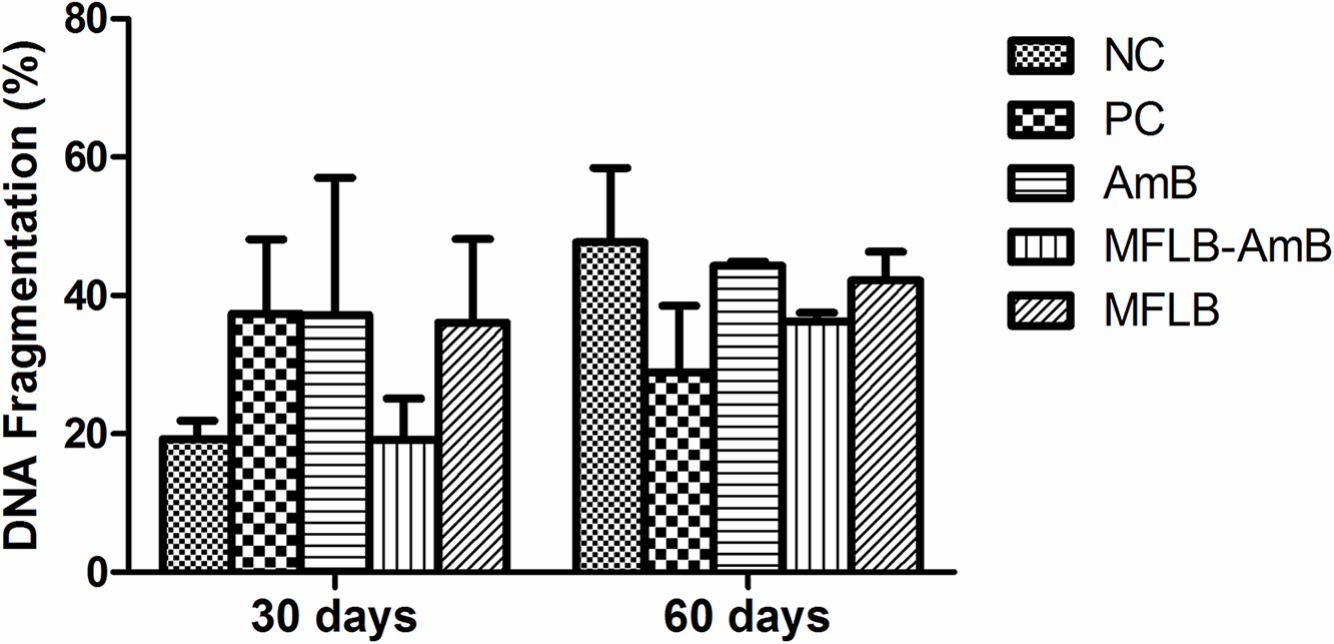
DNA fragmentation in bone marrow cells from mice treated with AmB, MFLB-AmB or MFLB. Female BALB/c mice uninfected (NC–negative control) and infected with *Paracoccidioides brasiliensis*, treated with PBS (PC–positive control), AmB (Amphotericin B), MFLB-AmB (nanosized magnetite surface-functionalized with a bilayer of lauric acid conjugated with Amphotericin B) and MFLB (nanosized magnetite surface-functionalized with a bilayer of lauric acid) for 30 or 60 days.

Treatment with the MFLB-AmB nanocomplex did not induce significant DNA fragmentation in the bone marrow cells of the mice (Fig 6). Additionally, no animals exhibited changes in appearance or losses of body weight during the treatments.

## Discussion

Our findings revealed that a newly developed MFLB-AmB nanocomplex was effective against an experimental model of paracoccidioidomycosis. The MFLB-AmB complex eliminated the fungal burden in the lungs and was less toxic than free (unbound) AmB both *in vitro* and *in vivo*.

Although free AmB was more effective against Pb18 with an MIC index of 0.25 μg/mL, the MFLB-AmB complex exhibited considerable antifungal activity with MIC index of 0.50 μg/mL (see Fig 1). No antifungal activity was observed at any MFLB concentrations tested. Additionally, the cytotoxicity of the MFLB-AmB nanocomplex in mesangial cells was significantly lower than that of the free AmB, indicating that when AmB is adsorbed onto the LA-bilayer-coated magnetite, the toxic side effect AmB toward mammalian urinary cells may be alleviated. Similarly, low toxicity of MFLB-AmB to mice peritoneal macrophages was observed. Cell viability subsequent to nanoparticle uptake is expected to be time-dependent. Initially, cells have to deal with the sudden exposure and internalization of nanoparticles, followed by a period of 6–10 hours of cellular growth as the cells resume their cellular activities, and the cell number either stabilizes or is reduced due to cell toxicity [32]. We found that free AmB and adsorbed AmB (MFLB-AmB) induced cytotoxicity similar to that of MFLB. Indeed, the enhanced uptake of surface-coated nanosized iron oxide particles by macrophages has previously been demonstrated [33, 34]. In this regard, cell uptake is linked to particle size, time dependence, and concentration dependence [33].

According to the literature, fungal cells are more susceptible to AmB than cultured animal cells. This characteristic is due to the specific interaction of AmB with ergosterol, a typical steroid found uniquely in the fungal cell wall, which leads to the formation of pores through the lipid membrane and consequent cell death [35–39]. Although AmB has a greater affinity for ergosterol, it also binds to the cholesterol found in mammalian cell membranes, which is believed to account for its toxicity in animals and humans, particularly renal cells [38, 40–42].

Although only free (unbound) AmB has been reported to bind to ergosterol [38], in the present study, we found that AmB adsorbed onto magnetic nanoparticles (MFLB-AmB) maintained its antifungal activity. Recently, Santos et al. [25] suggested that AmB grafted onto lauric acid (bilayer coated) pre-coated magnetite nanoparticles results in hydrophilic portions of AmB facing outwards, making AmB available to interact with the cell membrane forming the trans-membrane channels. While AmB incorporated into the MFLB-AmB nanocomplex exhibited an MIC index (0.50 μg/mL) that was two times higher than that of free AmB (0.25 μg/mL), the MIC value of MFLB-AmB nanocomplex is consistent with the MIC values of 0.03 to 1.0 μg/mL that have been reported in *in vitro* studies [43–45].

Indeed, engineering of nanosized drug carrier platforms based on iron oxide nanoparticles has attracted significant attention because these systems exhibit relatively low cell and tissue toxicities, are inexpensive to produce, are efficient, and immobilize biological materials at their surface [39]. Despite the good colloidal stability achieved by MF samples, it has been shown that a small fraction of the suspended magnetic particles tends to aggregate; however, aggregation can be reduced by various surface stabilizers [46]. Thus, the association of AmB with the surface pre-functionalized magnetic nanoparticles might represent an excellent material platform for the treatment of chronic forms of fungal disease [25]. In the present study, we used AmB associated with an LA-bilayer that coated magnetite (Fe_3_O_4_) nanoparticles. LA is known to stimulate the immune system by activating and releasing interleukin 2 [47]. Moreover, nanoparticles are known to enhance cellular uptake of the loaded drugs by various mechanisms, including protecting the drug from degradation. The efficacy of AmB is compromised by the high occurrences of adverse effects associated with the toxicity of the drug in humans. Therefore, we evaluated the cytotoxicity of the MFLB-AmB complex in mammalian cells via an MTT assay. We found that MFLB-AmB has relative low cytotoxicity, as demonstrated by higher LC_50_ values, in peritoneal macrophages and that the cytotoxicity was time and concentration dependent. However, the cytotoxicity of MFLB-AmB was similar to those of free AmB and MFLB.

Furthermore, morphological analyses of the livers, spleens, and kidneys of the infected animals treated with MFLB-AmB exhibited no tissue alterations. Biochemical tests are widely used to diagnose animal diseases and to monitor treatment responses. Literature indicates that treatment with free (unbound) AmB results in increased creatinine and urea in the serum due to renal vasoconstriction and a reduction of the glomerular filtration rate [48, 49]. Moreover, there are experimental data that suggest that free (unbound) AmB might influence the metabolic capacity of the liver [50]. Levels of certain liver enzymes in the blood can be indicative of liver injury. Under normal circumstances, these enzymes primarily reside within the cells of the liver, but when the liver is injured by any cause, these enzymes spill into the bloodstream. Among the most sensitive and widely used liver enzymes are the aminotransferases, including aspartate aminotransferase (AST) and alanine aminotransferase (ALT). Transferrin is also a plasma protein that is produced by liver and transports iron through the blood to the liver, spleen and bone marrow; controlling the level of free iron in biological fluids. The level of transferrin in the plasma decreases in conditions of liver and kidney diseases. Thus, we measured the serum levels of urea and creatinine as biomarkers of kidney functions and the levels of transferrin, AST, AST as biomarkers of liver injury. In the present study, we observed a lack of significant differences in all of these markers following treatment with MFLA-AmB, relative to the positive control (infected mice treated with PBS). These findings demonstrate the safety of our MFLA-AmB nanocomplex at the doses tested in this experiment.

Although its potential toxicity had previously been detected in clinical trials, free AmB in this study failed to elicit any cytotoxic effects in the kidneys, which might be related to the route of administration. Animals received intraperitoneal injections of free AmB daily for 30 or 60 days. This route was chosen to test for acute drug toxicity as the detoxifying effects of the entero-hepatic system would make the evaluation of a novel material via the gastrogavage route difficult. MFLB-AmB and MFLB were administered by nasal instillation because, compared to conventional oral therapy, inhalation drug delivery has been proven to be superior in terms of delivering high payloads of drug into the lungs [51]. Collectively our results demonstrate that the MFLB-AmB complex reduced the adverse effects and increased the therapeutic effects of AmB. Notably, the MFLB-AmB complex was administered to the animals every three days, while the free AmB was administered daily to the animals, similar to clinical trials.

Although morphology is a phenotypic aspect that is particularly important in toxicity studies, a more detailed analysis that allows for the early detection of the effects of a substance should be considered. Such early detection could involve the analysis of DNA fragmentation. Therefore, we also performed a genotoxicity test with bone marrow cells to determine whether MFLB-AmB induced DNA damage. Bone marrow tissue is easy to isolate and process, highly vascularized tissue and contains a population of rapidly cycling cells that is routinely used in DNA damage tests. The MFLB-AmB complex did not induce DNA fragmentation. These data reinforce those obtained from the histopathology analyses; i.e., the MFLB-AmB complex was toxic only to the cells of the fungus *P*. *brasiliensis*.

In both the human and the experimental models, cellular immunity is the primary defense mechanism against paracoccidioidomycosis [52]. Thus, we quantified cytokines, including interferon-gamma (IFN- γ), interleukin-4 (IL-4), 10 (IL-10), and 12 (IL-12), in the lungs. In a general, except for IL-10, all the other interleukin levels were reduced after 30 days of MFLB-Amb treatment. However, interleukin levels returned to the same as observed in uninfected animals after 60 days of MFLB-Amb treatment. It is interesting to note that it is exactly the opposite that occurs with the Amb animals group. Since both treatments (Amb and MFLB-Amb) successfully treated the paracoccidioidomycosis infection, it is possible that the decrease total dose of MFLB-AmB aided in decreasing the time for the levels of measured interleukins to return to the normal levels. Surely possible explanations exist for these results and futures studies are necessary to clarify this point.

According to Fortes and coworkers [31], increased levels of IL-10 and IL-4 are associated with the severe form of the disease, while IFN- γ and IL-12 are associated with mild forms of the disease. In the present study, mice treated with the MFLB-AmB nanocomplex lacked significant changes in IL-10 levels but reduced IL-4 after 30 days compared to the positive controls. These results are interesting because in humans the humoral immune response is not protective against paracoccidioidomycosis; rather, a response correlates with a poor prognoses for patients suffering from the disease. MFLB-AmB also reduced IL-12 and IFN-γ after 60 days compared to the positive control. During the course of a paracoccidioidomycosis infection, CD4^+^ lymphocytes synthesize IFN-γ and IL-12, which hinder the spread of the fungus. A possible explanation for both IFN-γ and IL-12 being low following MFLB-AmB is that treatment eliminated the fungal burden from the lungs; if there is no infection, the immune system does not need to synthesize these molecules.

In conclusion, our findings support the claim that when AmB is coupled to magnetic nanoparticles stabilized with bilayer lauric acid, as with our novel MFLB-AmB nanocomplex, AmB retains its efficacy against paracoccidioidomycosis without inducing acute hepatotoxic and nephrotoxic effects. Treatment in the form of MFLB-AmB also appears to allow for a reduction in the number of applications. Thus, this nanocomplex represents a promising alternative for the treatment of *Paracoccidioides brasiliensis*.
